# Influenza A virus in dairy cattle: infection biology and potential mammary gland-targeted vaccines

**DOI:** 10.1038/s41541-025-01063-7

**Published:** 2025-01-14

**Authors:** Rodrigo Prado Martins, Daniel Marc, Pierre Germon, Sascha Trapp, Ignacio Caballero-Posadas

**Affiliations:** 1 PFIE, INRAE, Nouzilly, France; 2 ISP, INRAE, Université de Tours, Nouzilly, France

**Keywords:** Viral infection, Vaccines

## Abstract

Influenza, a major “One Health” threat, has gained heightened attention following recent reports of highly pathogenic avian influenza in dairy cattle and cow-to-human transmission in the USA. This review explores general aspects of influenza A virus (IAV) biology, its interactions with mammalian hosts, and discusses the key considerations for developing vaccines to prevent or curtail IAV infection in the bovine mammary gland and its spread through milk.

## Introduction

Influenza A is a worldwide spread and highly contagious viral disease that ranks among the major public and veterinary health issues. Recent reports of infections by highly pathogenic avian influenza viruses (HPAIVs) in wild and domestic mammals worldwide^1,2^ point to an elevated risk of adaption of these viruses to mammalian hosts, which may ultimately lead to the emergence of a new pandemic strain. This threat has recently reached an unprecedented alarming level after the reports of sustained HPAIV (H5N1 clade 2.3.4.4b) infections in dairy cattle and cases of cow-to-human transmission in the USA^3–6^.

In this review, we provide a brief overview of the biology and natural history of influenza A virus (IAV) infections and describe the key mechanisms involved in the interactions between IAV and its mammalian hosts. We also highlight the main recent observations on HPAIV infections in dairy cows and discuss the major aspects to be considered for the development of candidate vaccines able to prevent productive IAV replication in the mammary gland and its spread through milk.

## Key features of Influenza A virus

IAV is the type species of the genus *Alphainfluenzavirus* and belongs to the Orthomyxoviridae family. Because of their rich diversity, IAVs are commonly classified as viral subtypes according to combinations of distinct serotypes of the two surface glycoproteins, haemagglutinin (HA or H, when referring to the serotype) and neuraminidase (NA or N), that decorate the viral envelope. Sixteen serotypes of HA and 9 serotypes of NA have been identified, mostly in avian influenza viruses (AIVs), and almost all of their possible combinations (HxNy) have been detected in field isolates. The viral genome, totalling about 13 kilobases, comprises 8 single-stranded RNAs of negative polarity. Both in the virion and in the infected cell, each of these genomic RNAs are associated with multiple copies of the viral nucleoprotein NP^7^, while the RNA termini are associated with the three subunits (PA, PB1, PB2) of the viral RNA-dependent RNA polymerase. Following HA binding to sialic acids (SAs) linked to cellular glycoproteins, the virus is internalized. After fusion of the viral envelope with the endosome membrane, its genetic material, in the form of eight ribonucleoproteins, is directed to the nucleus, where both viral mRNAs transcription and viral genome replication occur.

At the end of the viral cycle, 8 newly synthesized ribonucleoproteins (RNPs) associate with each other, along with the cellular membrane, leading to the budding of enveloped progeny virions. During this step, the segmented nature of the viral genome allows genomic reassortments, leading to the creation of viruses with new antigenic and phenotypic properties. Whenever a cell is infected by two distinct IAVs, their individual RNPs can be mixed in theoretically up to 256 possible viral genome combinations, including the two parental ones. Reassortments can be observed between two human IAVs (e.g. H1N1 and H3N2) infecting a single person. However, these events are far more common in wild aquatic and shore birds, which collectively constitute the main natural reservoir for avian influenza viruses (AIVs). The co-infection of a wild aquatic bird by two or more AIVs is not uncommon^8,9^, providing multiple opportunities for reassortment events.

## Expansion and diversification of H5N1 HPAIVs: impacts and trends

**Fig. 1:**
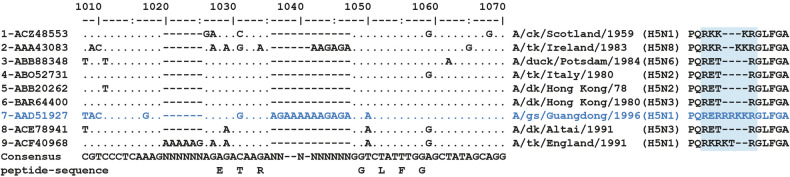
Alignment of HA nucleotide sequence (nucleotides 1008-1070 of the coding sequence). The peptide sequence corresponding to the cleavage site is shown on the right (blue rectangle highlights the stretch of basic residues upstream of the cleavage site). Alignment was performed using the “emma” and “shoawalign” tools in EMBOSS and manually edited. A few representative sequences from the Eurasian set of H5 sequences (up to year 1996) were retrieved from the Influenza virus resource. Sequences 1 and 9, corresponding to previous HPAIVs in Europe, were included for comparison.

H5N1 viruses belong to a long lineage of H5 AIVs that originated in China in the 1990s. The HA of all the current panzootic avian H5Nx viruses can be traced back to the progenitor virus A/goose/Guangdong/1996 (A/gs/Gd). This HA is the principal virulence determinant of these panzootic viruses, because of its particularly long polybasic cleavage site (PCS): **RERRRKKR** | GLFGA, which resulted from the insertion of untemplated purines by the “stuttering” viral polymerase (Fig. 1). The membrane fusion that occurs at the beginning of the viral cycle is mediated by the HA, once it is activated through cleavage into HA1 and HA2 by extracellular proteases acting at the N-terminus of the GLFGA peptide sequence. Due to the particularly long PCS of the HA of A/gs/Gd, this cleavage can be readily catalysed by ubiquitous proteases (such as furin), a prerequisite for the systemic dissemination of HPAIVs within their avian hosts. The HA of A/gs/Gd emerged from the Eurasian pool of low-pathogenicity avian H5 viruses^10^, most likely from H5N2 and H5N3 viruses that circulated in Asia at that time (Fig. 1). While the reassortment events between avian influenza viruses before 1996 were only poorly or not at all documented, the acquisition of this polybasic cleavage site was clearly the decisive factor in the surge of the current H5 HPAI viruses.

The major HPAI epizootic caused by A/gs/Gd and its descendants in Guangdong and Hong Kong in 1996–97 resulted in 18 human infections, of which 6 were lethal. This first epizootic was stopped, thanks to the culling of millions of poultry. However, “stamping-out” strategies failed to eliminate the pool of Asian H5 viruses. H5 HPAIVs expanded and diversified in the 1997–2002 period, infecting several species of wild and domestic birds^11^. From 2002 onwards, HPAIV belonging to the “genotype Z” caused several outbreaks in China and South East Asia. After these initial events at a regional scale, several important waves of H5 HPAI spread to other continents in the next two decades. In 2005–2008, Qinghai-lineage H5N1 reached Europe and Africa^11,12^, while a major wave of H5N8 viruses harbouring clade 2.3.4.4 H5 spread worldwide in 2014–2015^13^. This was followed in 2020 by the ongoing global wave of H5N1 viruses harbouring clade 2.3.4.4b H5^14,15^ and since 2022, more and more avian and mammalian species have been found to be infected by the currently circulating H5N1 viruses^16,17^.

## IAV infections in mammals: natural history and host response

Infections by IAVs have been detected in a wide array of avian and mammalian species. Wild migratory birds, especially waterfowl, are considered as the natural viral reservoir and play a role in the spread of IAV infections across all continents. Occasionally, these avian-origin IAVs, commonly referred to as AIVs, cross over from their natural reservoir to domestic birds, notably farmed chickens, turkeys, geese and ducks. In these hosts, some low-pathogenicity AIVs (LPAIVs) of the H5 or H7 types can evolve into HPAIVs through the above-mentioned insertion of untemplated purines. On the other hand, AIVs (LPAIVs or HPAIVs) in domestic birds can spill over to mammals including humans, causing sporadic or sustained infections. IAVs that are currently circulating in swine and in horses (H1N1, H1N2 and H3N2 strains in swine; H3N8 in horses) result from intricate combinations of spill-over and reassortment events that occurred in the preceding decades^18–20^. In humans, all seasonal influenza viruses that have been or that are currently circulating have likewise resulted from combinations of spill-over infections and virus reassortments. Such evolutionary events, often involving swine as an intermediate host, have caused the four influenza viruses pandemics of the 20th and 21st centuries: Spanish flu (H1N1) in 1918, Asian flu (H2N2) in 1957, Hong Kong flu (H3N2) in 1968 and the 2009 swine flu pandemic (H1N1/2009)^21^. Constantly circulating in the human population, human H3N2 and H1N1/2009 viruses are nowadays the cause of seasonal influenza epidemics (together with influenza B viruses of the B/Victoria and B/Yamagata lineages) which result in 290.000 to 645.000 deaths annually worldwide^22^. Sporadic infections by AIV without sustained intra-species transmission have been detected in numerous mammalian species including humans, pigs and various domestic or wild carnivores and marine mammals^23^. Sporadic spill-over infections leading to sustained intra-species transmission have so far only been reported in cats^24^, seals^25^, farmed fur animals (minks, foxes and racoon dogs)^26,27^ and, only recently, in cattle^3^.

Adaptation of AIVs to mammalian hosts requires amino acid substitutions in various viral proteins, with modifications in the HA protein and the viral polymerase playing a key role in this process. Certain substitutions in the HA protein can allow it to switch its receptor binding specificity, from avian-type SA-α2,3 (preferentially bound by AIVs) to mammalian-type SA-α2,6 (preferentially bound by human/mammalian IAVs)^28,29^. Beside several substitutions in HA that are required to allow airborne person-to-person transmission, human adaptation also involves substitution E627K in the PB2 subunit of the viral polymerase to enhance viral replication in mammalian cells^16,29^. While most H5 AIVs that have been isolated so far from milk or cattle have not acquired these well-known adaptation markers, they carry several other substitutions that may increase their transmissibility and virulence in mammals^30^.

Upon entry into the host cell via clathrin-mediated or clathrin- and caveolin-independent endocytic pathways^31^, IAV is recognized by at least 3 types of pattern recognition receptors (PRRs) from the innate immune system: toll-like receptors (TLRs) that bind dsRNA (TLR3) or ssRNA (TLR7 and TLR8), the retinoic acid-inducible gene I (RIG-I, recognizing 5’-triphosphate RNA) and the NOD-like receptor NLRP3 (a component of inflammasome)^32,33^. This recognition leads to the activation of the nuclear factor kappa B (NF-κB) and interferon-regulatory factors (IRFs), which trigger the expression of pro-inflammatory cytokines (IL-1β, IL-18, IL-6, IL-8, TNF-α among others) and type I and III interferons (IFN-α/β, IFN-λ). Activation of the IFNα receptor by type I IFNs triggers the JAK-STAT signalling cascade and the expression of interferon stimulated gene (ISGs) with potent antiviral activities^32–34^. Cytokine secretion will lead to the early recruitment of immune effector cells, such as NK cells, monocytes and neutrophils, with a later arrival and activation of lymphocytes and plasma cells^35^.

Immune responses against IAV infection are a double-edged sword. While a strong activation of innate immunity is needed to clear infection, a dysregulated response, especially in the case of human infections with certain AIVs, can initiate a so-called “cytokine storm”. This excessive cytokine secretion syndrome leads to severe and often fatal disease outcomes^36^. H5N1 HPAIV infections in humans often lead to pneumonia, acute respiratory distress syndrome (ARDS) and multi-organ failure, with a case fatality rate of approximately 50%. Patients show important lung inflammation, leukocyte infiltration, decreased peripheral lymphocyte count and high cytokine levels^37,38^.

Experimental infections of human volunteers with a seasonal human influenza virus (A/Texas/36/91 H1N1) demonstrated that the severity and magnitude of symptoms and viral replication correlate with the levels of IL-6 and IFN-α^39^. A similar correlation between the cytokine response and the severity of disease was observed using the ferret model of infection with seasonal influenza H1N1 and H3N2. While mild disease was associated with elevated levels of IFN-α and IFN-γ, severe disease was associated to elevated IL-6 levels^40^. In this model, infection with a H5N1 HPAIV produced a pathology similar to that observed following human H5N1 infections, including weight loss, lymphopenia, severe respiratory symptoms and death^41,42^. The key role of IFN type I signalling in the host defence to IAV has been confirmed in murine models based on animals deficient for IFNα receptor (*Ifnar−/−*) and STAT (*Stat−/−*). *Ifnar−/−* mice expressed decreased levels of interferon-stimulated genes (ISGs), while *Stat−/−* mice showed deficient viral clearance^43^. In addition, IFN-β deficient mice showed decreased survival rates and enhanced viral replication when infected with the SC35M strain (H7N7)^44^. Type I IFNs and dendritic cells help naïve CD8^+^ T cells to differentiate into cytotoxic T lymphocytes (CTLs) that target infected cells. Memory CD8^+^ T cells respond quickly to a second influenza infection but their efficacy decreases with time. Importantly, unlike neutralizing antibodies that are specific to a single HA serotype^45^, memory CD8 + T cells can potentially cross-react to different influenza subtypes since they recognize epitopes that are conserved in a variety of IAVs^46^.

## HPAIV outbreaks in bovine populations: general considerations and the unexpected role of the mammary gland

Unlike other livestock hosts at the human-animal interface (such as poultry and swine), bovine species have previously not been considered as susceptible targets for IAV infection, much less as potential reservoirs for continuous viral evolution^47^. Experimental infections of calves with HPAIV (H5N1 strain A/cat/Germany/R606/2006) via the intranasal route failed to induce clinical symptoms and resulted in low levels of viral shedding^48^. In addition, avian-type IAV receptors have previously been considered as absent from the upper and lower respiratory tract in cattle^49^. These observations, combined with data from multiple field studies^47^, suggested that the bovine species are less susceptible to infections with AIVs compared to other mammalian species expressing the avian-type SA-α2,3 receptor in the respiratory tract such as pigs and humans^50,51^. However, this understanding has been challenged by recent outbreaks of H5N1 clade 2.3.4.4b HPAIVs in multiple dairy cattle herds in the USA^3,5,52^. Two recent studies exploring the diversity of SA in bovine tissues have uncovered that both avian and mammalian-type IAV receptors are widely expressed in the respiratory tract and mammary gland of dairy cows and beef calves^53,54^. These observations demonstrate that the specific SA present in bovine tissues enable avian or mammalian-origin IAV to infect this species.

Since the initial detection in commercial dairy herds in Texas, USA, on March 2024, 924 confirmed cases of HPAI H5N1 clade 2.3.4.4b virus infection have been confirmed across 16 states as of January 11, 2025^52^. These infections are clinically characterized by nonspecific systemic symptoms including lethargy, reduced feed intake and rumen motility, as well as a sharp drop in milk production^3,55^. Although reports from the 1990-2000s have associated the presence IAVs or IAV-specific antibodies to a loss of milk production in bovine^56–58^, the recent cases of HPAIV infections are distinguished by a prominent involvement of the mammary gland in viral replication and spread within and between affected herds^3,55^. Infected animals show thick yellow milk with flecks and/or clots, often bacterial culture negative, indicating the occurrence of viral mastitis. Post-mortem analyses of affected cows confirmed the presence of neutrophilic mastitis concurrent with the presence of viral antigens in mammary alveolar epithelial cells and intraluminal cells^3^.

**Fig. 2:**
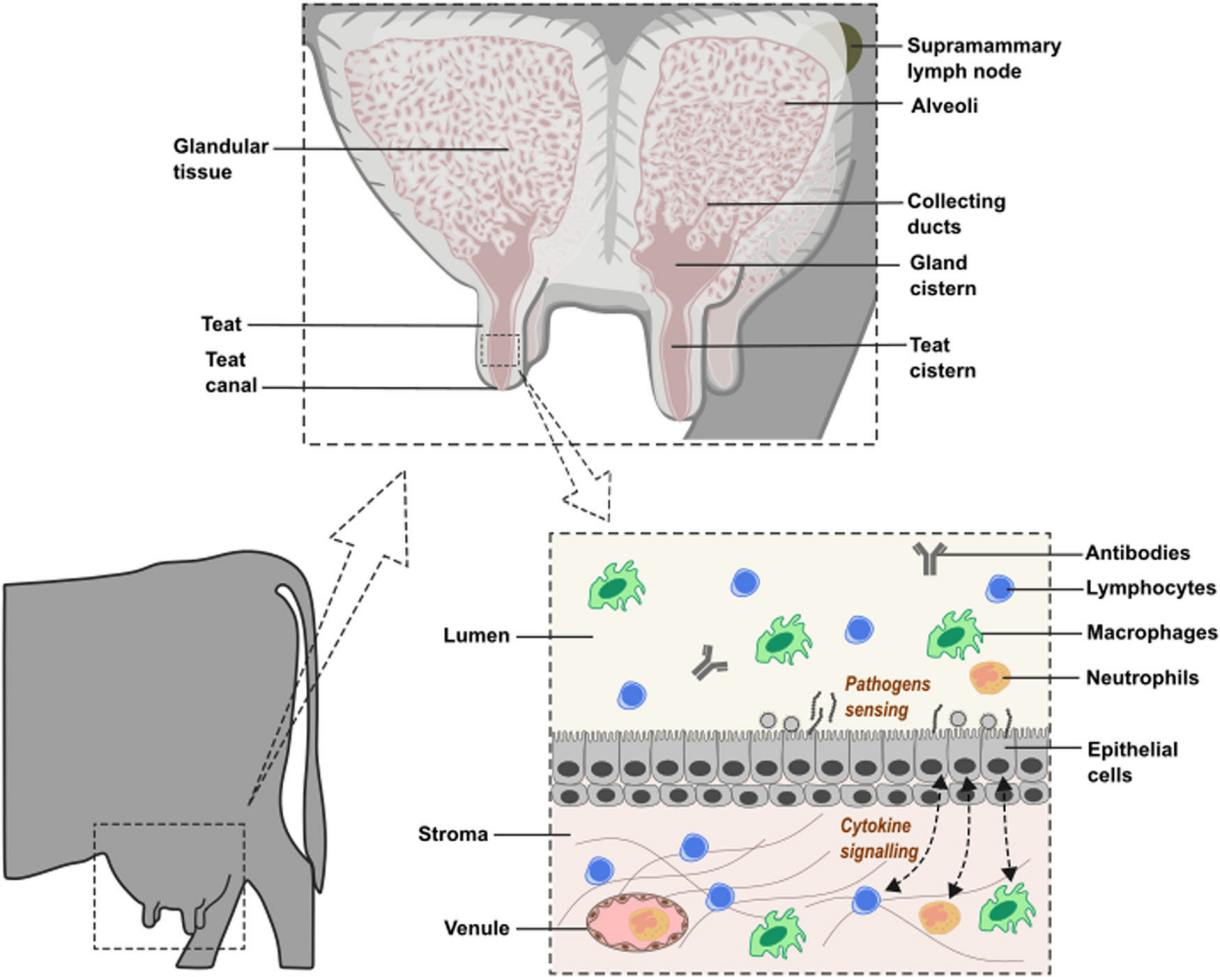
Anatomy of the bovine mammary gland and mediators of local immune response mechanisms. For details refer to^60^.

Mammary epithelial cells and leukocytes (macrophages, lymphocytes and neutrophils) present in numbers in the mammary gland act in concert by cytokine signalling to implement local innate and adaptive immune responses^59,60^ (Fig. 2). Upon IAV infection, these cells probably initiate immune mechanisms by the recognition of viral RNA and surface proteins. The expression of TLR7, a key endosomal receptor for the detection of viral RNA^61^, has not been demonstrated in the bovine mammary gland. However, the TLR1/TLR2 complex as well as the RIG-I receptor are reported to be expressed in the mammary gland^62–65^ and have been associated to viral detection^61^. Recognition of herpesviral motifs by the TLR1/TLR2 complex has been demonstrated in other species and the HA from measles virus was shown to activate murine and human antigen-presenting cells via the TLR2 receptor^66^. Therefore, the contribution of these receptors to viral recognition in the mammary gland shall not be overlooked and remains to be explored.

Virus-related mastitis has been mostly associated to diseases such as bovine viral leukemia^67^, foot and mouth disease^68^, bovine viral diarrhea and bovine herpesvirus-1 infections^69^, which predispose affected animals to secondary infections due to general immune suppression. Some evidence indicates that changes in the mammary gland and its secretion can be attributed to bacterial infections secondary to tissue damage caused by viruses^70,71^. Viruses can also infect the udder as a consequence of prolonged bacterial mastitis^72^ and, in some cases, multiply within the mammary gland without any clinical manisfestation^70,73^. Although viruses are not considered major udder pathogens^71,74^, intramammary inoculation experiments showed that the bovine mammary gland can act as a replication site for multiple viruses including bovine enterovirus and other viruses causing Newcastle disease, fowl plague, mumps, canine distemper, poliomyelitis, vesicular stomatitis, foot-and-mouth disease, infectious bovine rhinotracheitis, pseudo-cowpox, African swine fever, parainfluenza and human influenza^75–77^. These pioneer observations showed that large amounts of virus progeny can be produced in the bovine mammary gland and pointed to a potential role of milk in the transmission of viral diseases. Indeed, natural infections by bovine herpesviruses can result in intermittent shedding of the virus in milk by persistently affected animals^74^. Viral dissemination in milk has also been reported in cows infected with parainfluenza 3 virus^73^, foot and mouth disease virus^78^, bovine leukemia virus^79^ and recently, IAV^3^.

A genomic and epidemiologic investigation attributes the source of the multi-state cattle outbreak in the USA to a single transmission event from avian species^80^. Infections among cattle were reported to be horizontally transmitted by the displacement of lactating cows showing or not clinical disease^4,55^ but the precise mode of transmission and the initial site of virus replication remains unclear. Caserta et al. ^55^ hypothesized that HPAIVs could disseminate to organs such as the mammary gland via a short and low-level viremia upon oral or respiratory infection. The authors also proposed that infection could also occur through the teat orifice and cisternae, leading to viremia and virus dissemination to other tissues. To address this issue, an experimental infection study has been carried out to establish the role of the respiratory and intramammary routes in the onset of disease^81^. Viral replication in infected mammary gland was shown to be significantly greater than anticipated, reinforcing the notion that the udder and milk are primary sources of viral spread. Although experimental respiratory infection led to the detection of viable virus in only 2 out of 4 experimental animals, this route could be relevant for viral transmission in the context of animal facilities holding hundreds of animals^81^.

The establishment of endemic infections by HPAIVs in cattle could have significant implications from a One Health perspective. Evidence demonstrating multidirectional interspecies transmission of this pathogen suggests that, in this scenario, cattle and other animal species co-located on agricultural premises could act as mixing vessels facilitating the emergence of novel IAVs with enhanced zoonotic potential^3,80,81^. HPAIV dissemination in milk also represents a risk to individuals with occupational exposure to infected animals. Two cases of cow-to-human spread involving dairy farm workers have been reported. Both patients had mild illness with conjunctivitis and fully recovered^6^. As pasteurization inactivates influenza viruses^82,83^, the commercial milk supply is considered to be safe for consumption^84^. Nevertheless, raw milk and raw milk-based products from affected farms represent a risk for human consumers and other animals.

## Evaluating vaccine strategies to prevent HPAIV infections: focus on the bovine mammary gland

Efficient vaccines against bovine mastitis are still lacking^85^. However, the extensive trial-and-error efforts over the last decades to set up improved solutions have provided valuable insights into mammary gland immunity. This accumulated knowledge could contribute significantly to the design of candidate vaccines aimed at preventing viral proliferation in the bovine mammary gland and its subsequent dispersal via milk.

Vaccines against human seasonal influenza and swine, equine, or canine influenza are successfully used on a global scale since decades. Still, most of the current vaccines only protect against clinical disease and their effectiveness is often limited due to viral antigenic drift and resulting virus-vaccine mismatches^86^. Most of the licensed IAV vaccines (both in animals and humans) are inactivated vaccines that are mainly produced in embryonated chicken eggs. There are three types of inactivated influenza vaccines (IIV): whole virus vaccines (virus inactivation by formaldehyde or β-propiolactone treatment), split virus vaccines (virus disruption by physicochemical treatment) and subunit vaccines (preparations of the HA and NA viral surface proteins). These adjuvanted or non-adjuvanted vaccine formulations are predominantly applied via the intramuscular route. Live attenuated influenza vaccines (LAIV) are based on attenuated/non-virulent (often cold-adapted) viruses that were generated by serial passaging or reverse genetics. They are typically applied via the mucosal/intranasal route^87^.

Neutralizing antibodies directed against the HA globular head domain can provide sterilizing antiviral immunity and represent a well-established correlate of protection^88,89^. Antibodies directed against NA are likewise important as they can help limiting viral spread within the infected host^90^. IIV usually trigger strong IgG responses targeting both viral surface proteins, but their longevity is limited. Moreover, viral antigenic drift leading to virus-vaccine mismatches necessitates constant IIV reformulations that come with variable vaccine efficacies^91^. LAIV, mimicking natural viral infection, trigger less potent IgG responses than IIV, but they also induce robust mucosal (IgA secretion) and cellular (T-cell) immune responses, thus providing a more long lasting and potentially cross-protective (against antigenically distinct viruses) immunity^87,92^. These observations indicate that vaccine formulations capable of inducing not only a neutralizing IgG response, but also mucosal and cellular immune responses in the mammary gland might represent ideal candidates to limit symptoms and viral spread during HPAIV infections in cattle.

Polyreactive antibodies produced without prior antigenic stimulation, defined as natural antibodies (NAbs), are found in bovine serum and milk. These antibodies, primarily of the IgM isotype, bind to microbial molecules with low affinity and have been associated with improved defence against bacterial mastitis^93^. However, high titres of HPAIV in the mammary gland of infected cows^3,55^ suggests that local NAbs are insufficient to block viral replication. Bovine milk contains low levels of immunoglobulins (Igs), with a predominance of the IgG1 isotype. This particularity significantly influences bovine mammary immunity, as IgG1 is not opsonic for neutrophils in this species. To increase IgG2, IgA and IgM levels, and consequently enhance humoral and phagocytic responses in the mammary gland, local stimulation through natural infections or vaccination is required^94^. Baker et al. observed that the detection of virus-neutralizing antibodies in mammary quarters experimentally infected with HPAIV coincided with negative virus isolation^81^. This finding indicates that the induction of local influenza-specific antibodies might contribute to viral clearance from the udder, but further research is necessary to clarify the nature of involved Igs, the duration of this response, and importantly, if it can be modulated through vaccination.

Different immunisation approaches have enabled the production of antibodies targeting antigens from mastitis-causing bacteria in serum, but achieving protective antibody responses at sufficient levels in the mammary gland remains a challenging task. Prime and booster subcutaneous (SC) injections of an oil-in-water adjuvanted subunit vaccine in the mammary gland suspensory ligament have been shown to induce higher vaccine-specific IgG1 levels in milk, but not IgG2 or IgA levels, compared to two intramuscular (IM) administrations in the neck or an intramammary (IMM) prime followed by an SC booster in the mammary gland ligament^95^. An increase in vaccine-specific IgG1, but not IgG2, in milk has also been reported following prime and booster SC injections in the neck of a vaccine composed of recombinant antigens combined with Emulsigen^®^-D^96^. Another study demonstrated that neither IM/IM nor IM/IMM prime and boost immunisations with killed *Escherichia coli* emulsified in oil adjuvant (Montanide ISA 61VG®) or *E. coli* culture supernatant improve milk bactericidal and opsonic activity^97^, probably due to insufficient IgG2 levels, a limiting factor for bacterial clearance by neutrophils. Vaccination-induced IgG1 in the mammary gland could contribute to viral elimination by increasing the capacities of macrophages to act as phagocytic and antigen presenting cells. This Ig isotype represents the majority of IgG antibodies targeting IAV and is highly effective at direct virus inhibition^91^. Nevertheless, IgA are considered to present the highest affinity for influenza HA^91^ and this isotype is present at low levels in bovine milk^94^. Immunisations with *S. aureus* α-toxoid combined with different adjuvants showed that alum supplemented with both saponin and oil result in higher IgG1, IgG2 and IgA titres in milk when compared to alum–oil or alum–saponin^98^.

Evidence suggests that adaptive cell-mediated immunity (CMI) also confers host resistance to influenza. A study carried out in a natural infection setting uncovered that a higher frequency of cross-reactive cytotoxic T-cells (CTL; CD8^+^IFN-γ^+^IL-2^−^) is associated with reduced illness severity and absence of viral shedding in healthy human individuals infected with H1N1pdm09^99^. In another report exploring pre-infection cellular components related with asymptomatic and symptomatic influenza illness in vaccinated and unvaccinated adult individuals, it has been revealed that protection from infection is associated with increased frequency of polyfunctional CD4^+^ and CD8^+^ T cells, circulating T follicular helper, myeloid dendritic cells, Th17 cells and innate effector CD16-expressing cytotoxic and cytokine-producing NK cells^100^.

Pre-clinical evaluation of virus-vectored candidate vaccines indicate that the presence of CTL^91^ and CD4^+^^92^ T-cells recognizing highly conserved influenza proteins generates heterosubtypic immunity across IAV strains. T-cell-inducing vaccines could therefore counterbalance IAV escape mechanisms to circumvent humoral immunity. Of note, the direct activity of antigen-specific T-cells, rather than antibodies, has been reported as a major mechanism for controlling and preventing infections in the mammary gland^97,101^. These observations suggest that the capacity to efficiently trigger local CMI mechanisms should be incorporated into the requirements of a candidate vaccine designed to prevent HPAIV infections in the bovine mammary gland.

A recent study demonstrated that mucosal immunization via the nasal route outperforms IM immunization in conferring cross-protection against influenza in mice^102^. The authors also highlighted that influenza cross-protection can be bolstered by heterologous sequential immunizations combining different administration routes and these findings have been attributed to improved IFN-γ-mediated effector functions and T-cells avidity^102^. Interestingly, local immunization has also been reported to modify favourably the course of infection in the mammary gland by limiting inflammation and accelerating bacterial clearance. Heterologous immunisation against *E. coli* in an IM/IMM prime/booster regime resulted in earlier and higher IFN-γ production in milk upon IMM infectious challenge^97^. Further investigation suggested that intramammary immunizations are pivotal for the establishment of resident memory T-cells in the bovine mammary tissue^103^. In this sense, an immunization strategy taking advantage of the intramammary route could promote the elimination of HPAIV at the site of entry in case of intramammary transmission and/or prevent productive viral replication in the mammary gland and dispersal in milk. Nevertheless, the development of an intramammary vaccine candidate against HPAIV necessitates careful consideration in the design of formulations, including the selection of appropriate adjuvants, to avoid compromising the role of the mammary gland as a milk-producing organ.

**Fig. 3:**
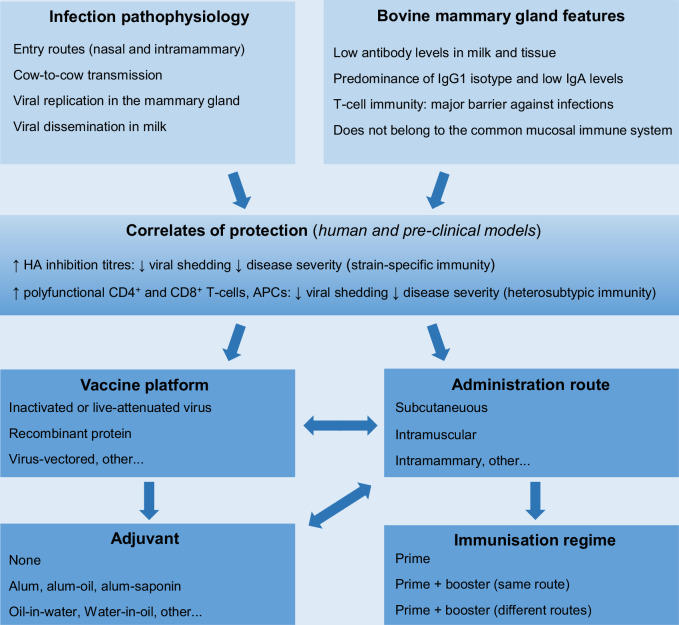
Key considerations for designing vaccines against HPAIV infections in the bovine mammary gland. APC antigen presenting cells, HA haemagglunitin.

Intranasal immunisation with an oil-in-water adjuvanted subunit vaccine failed to elicit measurable antibody responses in dairy cattle and this outcome has been attributed to the inability of recombinant proteins to stimulate bovine immune responses at the nasal mucosa^104^. In contrast, intranasal vaccination with live formulations has been reported to provide benefits in preventing bovine respiratory diseases that include achieving full efficacy in the presence of maternally derived antibodies and stimulating the common mucosal immune system (CMIS)^105^. In this context, induction of mucosal immunity at the respiratory tract by vaccination could play a role in reducing airborne transmission of HPAIV. However, multiple observations suggest that the mammary gland of dairy ruminants does not belong to the CMIS^101^. This particularity represents a limitation for strategies based on oral or intranasal immunisations that simultaneously target the mammary gland. These and other factors involved in designing a vaccine candidate targeting the mammary gland are outlined in Fig. 3.

## Conclusions

Controlling HPAIV infections in cattle represents a major “One Health” emergency. Improvements in biosecurity and hygiene measures, coupled to a “cull plus vaccination” strategy, have proven to be efficient in controlling successive H5 HPAI epizootics in domestic birds^106^. For this approach to be applied to outbreaks in cattle, the development of efficient vaccines will be crucial. Although the role of the mammary gland in the pathophysiology of HPAIV infections in the bovine species needs to be better understood, the observed viral tropism for mammary epithelial cells strongly suggests that a successful vaccine candidate must induce robust anti-viral protective responses in the udder. The limited advances in the implementation of efficient vaccines for mastitis in cattle over the past decades^85^ indicate that this task represents a significant challenge and collaborative efforts among teams from the fields of virology, vaccinology and bovine immunology will be essential to achieve this objective.

## References

[1] Venkatesan, P. Avian influenza spillover into mammals. *Lancet Microbe* **4**, e492 (2023). 37247629 10.1016/S2666-5247(23)00173-8

[2] European Food Safety, A. et al. Avian influenza overview March-June 2024. *EFSA J.* **22**, e8930 (2024). 39036773 10.2903/j.efsa.2024.8930 PMC11258884

[3] Burrough, E. R. et al. Highly pathogenic avian influenza A(H5N1) clade 2.3.4.4b virus infection in domestic dairy cattle and cats, United States, 2024. *Emerg. Infect. Dis.* **30**, 1335–1343 (2024). 38683888 10.3201/eid3007.240508 PMC11210653

[4] USDA-APHIS. Highly pathogenic avian influenza H5N1 genotype B3.13 in dairy cattle: national epidemiologic brief. 2024 08/06/2024 [cited 2024 01/08/2024]Available from: https://www.aphis.usda.gov/sites/default/files/hpai-dairy-national-epi-brief.pdf.

[5] Oguzie, J. U. et al. Avian influenza A(H5N1) virus among dairy cattle, Texas, USA. *Emerg. Infect. Dis.* **30**, 1425–1429 (2024). 38848249 10.3201/eid3007.240717 PMC11210641

[6] Garg, S. et al. Outbreak of highly pathogenic avian influenza A(H5N1) viruses in U.S. dairy cattle and detection of two human cases - United States, 2024. *MMWR Morb. Mortal. Wkly Rep.* **73**, 501–505 (2024). 38814843 10.15585/mmwr.mm7321e1 PMC11152367

[7] Lee, N. et al. Genome-wide analysis of influenza viral RNA and nucleoprotein association. *Nucleic Acids Res.* **45**, 8968–8977 (2017). 28911100 10.1093/nar/gkx584 PMC5587783

[8] Wang, R. et al. Examining the hemagglutinin subtype diversity among wild duck-origin influenza A viruses using ethanol-fixed cloacal swabs and a novel RT-PCR method. *Virology* **375**, 182–189 (2008). 18308356 10.1016/j.virol.2008.01.041 PMC2397557

[9] Dugan, V. G. et al. The evolutionary genetics and emergence of avian influenza viruses in wild birds. *PLoS Pathog.* **4**, e1000076 (2008). 18516303 10.1371/journal.ppat.1000076 PMC2387073

[10] Lee, D. H., Criado, M. F. & Swayne, D. E. Pathobiological origins and evolutionary history of highly pathogenic avian influenza viruses. *Cold Spring Harb. Perspect. Med.* **11**, a038679 (2021). 31964650 10.1101/cshperspect.a038679 PMC7849344

[11] Sonnberg, S., Webby, R. J. & Webster, R. G. Natural history of highly pathogenic avian influenza H5N1. *Virus Res.* **178**, 63–77 (2013). 23735535 10.1016/j.virusres.2013.05.009 PMC3787969

[12] Verhagen, J. H., Fouchier, R. A. M. & Lewis, N. Highly pathogenic avian influenza viruses at the wild-domestic bird interface in Europe: future directions for research and surveillance. *Viruses* **13**, 212 (2021). 33573231 10.3390/v13020212 PMC7912471

[13] Global Consortium for, H.N. & Related Influenza, V. Role for migratory wild birds in the global spread of avian influenza H5N8. *Science* 354, 213–217 (2016). 10.1126/science.aaf8852 PMC5972003 27738169

[14] Mirolo, M. et al. Highly pathogenic avian influenza A virus (HPAIV) H5N1 infection in two European grey seals (Halichoerus grypus) with encephalitis. *Emerg. Microbes Infect.* **12**, e2257810 (2023). 37682060 10.1080/22221751.2023.2257810 PMC10768861

[15] Xie, R. et al. The episodic resurgence of highly pathogenic avian influenza H5 virus. *Nature* **622**, 810–817 (2023). 37853121 10.1038/s41586-023-06631-2

[16] Plaza, P. I., Gamarra-Toledo, V., Eugui, J. R. & Lambertucci, S. A. Recent changes in patterns of mammal infection with highly pathogenic avian influenza A(H5N1) virus worldwide. *Emerg. Infect. Dis.* **30**, 444–452 (2024). 38407173 10.3201/eid3003.231098 PMC10902543

[17] Neumann, G. & Kawaoka, Y. Highly pathogenic H5N1 avian influenza virus outbreak in cattle: the knowns and unknowns. *Nat. Rev. Microbiol.* **22**, 525–526 (2024). 39060613 10.1038/s41579-024-01087-1 PMC12720498

[18] Simon, G. et al. European surveillance network for influenza in pigs: surveillance programs, diagnostic tools and Swine influenza virus subtypes identified in 14 European countries from 2010 to 2013. *PLoS ONE* **9**, e115815 (2014). 25542013 10.1371/journal.pone.0115815 PMC4277368

[19] Vincent, A. et al. Review of influenza A virus in swine worldwide: a call for increased surveillance and research. *Zoonoses Public Health* **61**, 4–17 (2014). 23556412 10.1111/zph.12049

[20] Bravo-Vasquez, N. et al. Equine-Like H3 avian influenza viruses in wild birds, Chile. *Emerg. Infect. Dis.* **26**, 2887–2898 (2020). 33219648 10.3201/eid2612.202063 PMC7706983

[21] Krammer, F. et al. Influenza. *Nat. Rev. Dis. Prim.* **4**, 3 (2018). 29955068 10.1038/s41572-018-0002-y PMC7097467

[22] Iuliano, A. D. et al. Estimates of global seasonal influenza-associated respiratory mortality: a modelling study. *Lancet* **391**, 1285–1300 (2018). 29248255 10.1016/S0140-6736(17)33293-2 PMC5935243

[23] Abdelwhab, E. M. & Mettenleiter, T. C. Zoonotic animal influenza virus and potential mixing vessel hosts. *Viruses* **15**, 980 (2023). 10.3390/v15040980 PMC10145017 37112960

[24] Lee, C. T. et al. Outbreak of influenza A(H7N2) among cats in an animal shelter with cat-to-human transmission-New York City, 2016. *Clin. Infect. Dis.* **65**, 1927–1929 (2017). 29020187 10.1093/cid/cix668

[25] Bodewes, R. et al. Avian influenza A(H10N7) virus-associated mass deaths among harbor seals. *Emerg. Infect. Dis.* **21**, 720–722 (2015). 25811303 10.3201/eid2104.141675 PMC4378483

[26] Aguero, M. et al. Highly pathogenic avian influenza A(H5N1) virus infection in farmed minks, Spain, October 2022. *Eur. Surveill.* **28**, 2300001 (2023). 10.2807/1560-7917.ES.2023.28.3.2300001 PMC9853945 36695488

[27] Lindh, E. et al. Highly pathogenic avian influenza A(H5N1) virus infection on multiple fur farms in the South and Central Ostrobothnia regions of Finland, July 2023. *Eur. Surveill.* **28**, 2300400 (2023). 10.2807/1560-7917.ES.2023.28.31.2300400 PMC10401912 37535475

[28] Kuchipudi, S. V. et al. Sialic acid receptors: the key to solving the enigma of zoonotic virus spillover. *Viruses* **13**, 262 (2021). 33567791 10.3390/v13020262 PMC7915228

[29] Thompson, A. J. & Paulson, J. C. Adaptation of influenza viruses to human airway receptors. *J. Biol. Chem.* **296**, 100017 (2021). 33144323 10.1074/jbc.REV120.013309 PMC7948470

[30] Gu, C. et al. A human isolate of bovine H5N1 is transmissible and lethal in animal models. *Nature* **636**, 711–718 (2024). 10.1038/s41586-024-08254-7 PMC12629513 39467571

[31] Rust, M. J., Lakadamyali, M., Zhang, F. & Zhuang, X. Assembly of endocytic machinery around individual influenza viruses during viral entry. *Nat. Struct. Mol. Biol.* **11**, 567–573 (2004). 15122347 10.1038/nsmb769 PMC2748740

[32] Iwasaki, A. & Pillai, P. S. Innate immunity to influenza virus infection. *Nat. Rev. Immunol.* **14**, 315–328 (2014). 24762827 10.1038/nri3665 PMC4104278

[33] Biondo, C., Lentini, G., Beninati, C. & Teti, G. The dual role of innate immunity during influenza. *Biomed. J.* **42**, 8–18 (2019). 30987709 10.1016/j.bj.2018.12.009 PMC6468094

[34] Guo, X. J. & Thomas, P. G. New fronts emerge in the influenza cytokine storm. *Semin. Immunopathol.* **39**, 541–550 (2017). 28555383 10.1007/s00281-017-0636-y PMC5580809

[35] Taubenberger, J. K. & Morens, D. M. The pathology of influenza virus infections. *Annu. Rev. Pathol.* **3**, 499–522 (2008). 18039138 10.1146/annurev.pathmechdis.3.121806.154316 PMC2504709

[36] Jiang, H. & Zhang, Z. Immune response in influenza virus infection and modulation of immune injury by viral neuraminidase. *Virol. J.* **20**, 193 (2023). 37641134 10.1186/s12985-023-02164-2 PMC10463456

[37] Uiprasertkul, M. et al. Apoptosis and pathogenesis of avian influenza A (H5N1) virus in humans. *Emerg. Infect. Dis.* **13**, 708–712 (2007). 17553248 10.3201/eid1305.060572 PMC2738443

[38] de Jong, M. D. et al. Fatal outcome of human influenza A (H5N1) is associated with high viral load and hypercytokinemia. *Nat. Med.* **12**, 1203–1207 (2006). 16964257 10.1038/nm1477 PMC4333202

[39] Hayden, F. G. et al. Local and systemic cytokine responses during experimental human influenza A virus infection. Relation to symptom formation and host defense. *J. Clin. Invest.* **101**, 643–649 (1998). 9449698 10.1172/JCI1355 PMC508608

[40] Svitek, N., Rudd, P. A., Obojes, K., Pillet, S. & von Messling, V. Severe seasonal influenza in ferrets correlates with reduced interferon and increased IL-6 induction. *Virology* **376**, 53–59 (2008). 18420248 10.1016/j.virol.2008.02.035

[41] Maines, T. R. et al. Avian influenza (H5N1) viruses isolated from humans in Asia in 2004 exhibit increased virulence in mammals. *J. Virol.* **79**, 11788–11800 (2005). 16140756 10.1128/JVI.79.18.11788-11800.2005 PMC1212624

[42] Restori, K. H. et al. Risk assessment of a highly pathogenic H5N1 influenza virus from mink. *Nat. Commun.* **15**, 4112 (2024). 38750016 10.1038/s41467-024-48475-y PMC11096306

[43] Davidson, S., Crotta, S., McCabe, T. M. & Wack, A. Pathogenic potential of interferon alphabeta in acute influenza infection. *Nat. Commun.* **5**, 3864 (2014). 24844667 10.1038/ncomms4864 PMC4033792

[44] Koerner, I., Kochs, G., Kalinke, U., Weiss, S. & Staeheli, P. Protective role of beta interferon in host defense against influenza A virus. *J. Virol.* **81**, 2025–2030 (2007). 17151098 10.1128/JVI.01718-06 PMC1797552

[45] Chen, X. et al. Host immune response to influenza A virus infection. *Front. Immunol.* **9**, 320 (2018). 29556226 10.3389/fimmu.2018.00320 PMC5845129

[46] Grant, E. J., Quinones-Parra, S. M., Clemens, E. B. & Kedzierska, K. Human influenza viruses and CD8(+) T cell responses. *Curr. Opin. Virol.* **16**, 132–142 (2016). 26974887 10.1016/j.coviro.2016.01.016

[47] Sreenivasan, C. C., Thomas, M., Kaushik, R. S., Wang, D. & Li, F. Influenza A in bovine species: a narrative literature review. *Viruses* **11**, 561 (2019). 31213032 10.3390/v11060561 PMC6631717

[48] Kalthoff, D., Hoffmann, B., Harder, T., Durban, M. & Beer, M. Experimental infection of cattle with highly pathogenic avian influenza virus (H5N1). *Emerg. Infect. Dis.* **14**, 1132–1134 (2008). 18598640 10.3201/eid1407.071468 PMC2600352

[49] Thontiravong, A., Kitikoon, P., Oraveerakul, K., Poovorawan, Y., & Rung-ruangkijkrai, T. Influenza A virus receptor identification in the respiratory tract of quail, pig, cow and swamp buffalo. *Thai J. Vet. Med.* **41**, 6 (2011).

[50] Nelli, R. K. et al. Comparative distribution of human and avian type sialic acid influenza receptors in the pig. *BMC Vet. Res.* **6**, 4 (2010). 20105300 10.1186/1746-6148-6-4 PMC2832630

[51] Shinya, K. et al. Avian flu: influenza virus receptors in the human airway. *Nature* **440**, 435–436 (2006). 16554799 10.1038/440435a

[52] USDA-APHIS. HPAI confirmed cases in livestock. 2025 11/01/2025 [cited 2025 11/01/2025]. Available from: https://www.aphis.usda.gov/livestock-poultry-disease/avian/avian-influenza/hpai-detections/hpai-confirmed-cases-livestock.

[53] Nelli, R. K. et al. Sialic acid receptor specificity in mammary gland of dairy cattle infected with highly pathogenic avian influenza A(H5N1) virus. *Emerg. Infect. Dis.* **30**, 1361–1373 (2024). 38861554 10.3201/eid3007.240689 PMC11210646

[54] Kristensen, C., Larsen, L. E., Trebbien, R. & Jensen, H. E. The avian influenza A virus receptor SA-alpha2,3-Gal is expressed in the porcine nasal mucosa sustaining the pig as a mixing vessel for new influenza viruses. *Virus Res.* **340**, 199304 (2024). 38142890 10.1016/j.virusres.2023.199304 PMC10793167

[55] Caserta, L. C. et al. Spillover of highly pathogenic avian influenza H5N1 virus to dairy cattle. *Nature* **634**, 669–676 (2024). 10.1038/s41586-024-07849-4 PMC11485258 39053575

[56] Gunning, R. F., Brown, I. H. & Crawshaw, T. R. Evidence of influenza A virus infection in dairy cows with sporadic milk drop syndrome. *Vet. Rec.* **145**, 556–557 (1999). 10609575 10.1136/vr.145.19.556

[57] Brown, I. H., Crawshaw, T. R., Harris, P. A. & Alexander, D. J. Detection of antibodies to influenza A virus in cattle in association with respiratory disease and reduced milk yield. *Vet. Rec.* **143**, 637–638 (1998). 9881443

[58] Crawshaw, T. R., Brown, I. H., Essen, S. C. & Young, S. C. Significant rising antibody titres to influenza A are associated with an acute reduction in milk yield in cattle. *Vet. J.* **178**, 98–102 (2008). 17851097 10.1016/j.tvjl.2007.07.022

[59] Riollet, C., Rainard, P. & Poutrel, B. Cells and cytokines in inflammatory secretions of bovine mammary gland. *Adv. Exp. Med. Biol.* **480**, 247–258 (2000). 10959433 10.1007/0-306-46832-8_30

[60] Rainard, P., Gilbert, F. B. & Germon, P. Immune defenses of the mammary gland epithelium of dairy ruminants. *Front. Immunol.* **13**, 1031785 (2022). 36341445 10.3389/fimmu.2022.1031785 PMC9634088

[61] Brubaker, S. W., Bonham, K. S., Zanoni, I. & Kagan, J. C. Innate immune pattern recognition: a cell biological perspective. *Annu. Rev. Immunol.* **33**, 257–290 (2015). 25581309 10.1146/annurev-immunol-032414-112240 PMC5146691

[62] Ibeagha-Awemu, E. M. et al. Bacterial lipopolysaccharide induces increased expression of toll-like receptor (TLR) 4 and downstream TLR signaling molecules in bovine mammary epithelial cells. *Vet. Res.* **39**, 11 (2008). 18096120 10.1051/vetres:2007047

[63] Petzl, W. et al. *Escherichia coli*, but not *Staphylococcus aureus* triggers an early increased expression of factors contributing to the innate immune defense in the udder of the cow. *Vet. Res.* **39**, 18 (2008). 18258172 10.1051/vetres:2007057

[64] Porcherie, A. et al. Repertoire of *Escherichia coli* agonists sensed by innate immunity receptors of the bovine udder and mammary epithelial cells. *Vet. Res.* **43**, 14 (2012). 22330199 10.1186/1297-9716-43-14 PMC3305352

[65] Buitenhuis, B., Rontved, C. M., Edwards, S. M., Ingvartsen, K. L. & Sorensen, P. In depth analysis of genes and pathways of the mammary gland involved in the pathogenesis of bovine Escherichia coli-mastitis. *BMC Genom.* **12**, 130 (2011). 10.1186/1471-2164-12-130 PMC3053262 21352611

[66] Bieback, K. et al. Hemagglutinin protein of wild-type measles virus activates toll-like receptor 2 signaling. *J. Virol.* **76**, 8729–8736 (2002). 12163593 10.1128/JVI.76.17.8729-8736.2002 PMC136986

[67] Watanabe, A. et al. Association between bovine leukemia virus proviral load and severity of clinical mastitis. *J. Vet. Med. Sci.* **81**, 1431–1437 (2019). 31406037 10.1292/jvms.19-0285 PMC6863728

[68] Lewis, R. A., Kashongwe, O. B. & Bebe, B. O. Quantifying production losses associated with foot and mouth disease outbreaks on large-scale dairy farms in Rift valley, Kenya. *Trop. Anim. Health Prod.* **55**, 293 (2023). 37608201 10.1007/s11250-023-03707-z

[69] Barkema, H. W., Green, M. J., Bradley, A. J. & Zadoks, R. N. Invited review: the role of contagious disease in udder health. *J. Dairy Sci.* **92**, 4717–4729 (2009). 19762787 10.3168/jds.2009-2347 PMC2765761

[70] Burrows, R., Mann, J. A., Greig, A., Chapman, W. G. & Goodridge, D. The growth and persistence of foot-and-mouth disease virus in the bovine mammary gland. *J. Hyg.* **69**, 307–321 (1971). 4326249 10.1017/s0022172400021537 PMC2130882

[71] Wellenberg, G. J., van der Poel, W. H. & Van Oirschot, J. T. Viral infections and bovine mastitis: a review. *Vet. Microbiol.* **88**, 27–45 (2002). 12119136 10.1016/s0378-1135(02)00098-6

[72] Kalman, D., Janosi, S. & Egyed, L. Role of bovine herpesvirus 4 in bacterial bovine mastitis. *Microb. Pathog.* **37**, 125–129 (2004). 15351035 10.1016/j.micpath.2004.06.011

[73] Kawakami, Y. et al. Infection of cattle with parainfluenza 3 virus with special reference to udder infection. I. Virus isolation from milk. *Jpn J. Microbiol.* **10**, 159–169 (1966). 4290964 10.1111/j.1348-0421.1966.tb00304.x

[74] Herlekar, D. A., Shashikant, C. S., Gurjar, A. A. & Jayarao, B. M. Presence of viral and bacterial organisms in milk and their association with somatic cell counts. *J. Dairy Sci.* **96**, 6336–6346 (2013). 23972495 10.3168/jds.2013-6631

[75] Kawakami, Y. et al. Infection of cattle with parainfluenza 3 virus with special reference to udder infection. II. Pathogenicity of the virus for cattle, with particular reference to the mammary gland. *Jpn J. Microbiol.* **10**, 171–182 (1966). 4290965 10.1111/j.1348-0421.1966.tb00305.x

[76] Mitchell, C. A., Walker, R. V. & Bannister, G. L. Further experiments relating to the propagation of virus in the bovine mammary gland. *Can. J. Comp. Med. Vet. Sci.* **17**, 218–222 (1953). 17648631 PMC1791540

[77] Afshar, A. B. & Viral, G. L. infections of the bovine mammary gland. *Vet. Bull.* **40**, 6 (1970).

[78] Paton, D. J., Gubbins, S. & King, D. P. Understanding the transmission of foot-and-mouth disease virus at different scales. *Curr. Opin. Virol.* **28**, 85–91 (2018). 29245054 10.1016/j.coviro.2017.11.013

[79] Marawan, M. A. et al. Bovine leukaemia virus: current epidemiological circumstance and future prospective. *Viruses* **13**, 2167 (2021). 10.3390/v13112167 PMC8618541 34834973

[80] Nguyen, T.-Q. et al. Emergence and interstate spread of highly pathogenic avian influenza A(H5N1) in dairy cattle. *bioRxiv*, 2024.05.01.591751 (2024). 10.1126/science.adq0900 40273240

[81] Baker, A. L. et al. Experimental reproduction of viral replication and disease in dairy calves and lactating cows inoculated with highly pathogenic avian influenza H5N1 clade 2.3.4.4b. *bioRxiv*, 2024.07.12.603337 (2024).

[82] Cui, P. et al. Does pasteurization inactivate bird flu virus in milk? *Emerg. Microbes Infect.* **13**, 2364732 (2024). 38832658 10.1080/22221751.2024.2364732 PMC11182070

[83] Palme, D. I., Lang, J., Helke, D., Kuryshko, M. & Abdelwhab, E. M. Strain-dependent variations in replication of European clade 2.3.4.4b influenza A(H5N1) viruses in bovine cells and thermal inactivation in semi-skimmed or whole milk. *Eur. Surveill.* **29**, 2400436 (2024). 10.2807/1560-7917.ES.2024.29.30.2400436 PMC11274848 39056199

[84] Spackman, E. et al. Characterization of highly pathogenic avian influenza virus in retail dairy products in the US. *J. Virol.* **98**, e0088124 (2024). 38958444 10.1128/jvi.00881-24 PMC11264905

[85] Rainard, P., Gilbert, F. B., Martins, R. P., Germon, P. & Foucras, G. Progress towards the elusive mastitis vaccines. *Vaccines* **10**, 296 (2022). 35214754 10.3390/vaccines10020296 PMC8876843

[86] Tripp, R. A. Understanding immunity to influenza: implications for future vaccine development. *Expert Rev. Vaccines* **22**, 871–875 (2023). 37794732 10.1080/14760584.2023.2266033

[87] Yamayoshi, S. & Kawaoka, Y. Current and future influenza vaccines. *Nat. Med.* **25**, 212–220 (2019). 30692696 10.1038/s41591-018-0340-z PMC12973209

[88] Becker, T., Elbahesh, H., Reperant, L. A., Rimmelzwaan, G. F. & Osterhaus, A. Influenza vaccines: successes and continuing challenges. *J. Infect. Dis.* **224**, S405–S419 (2021). 34590139 10.1093/infdis/jiab269 PMC8482026

[89] Bean, R. et al. Mucosal correlates of protection after influenza viral challenge of vaccinated and unvaccinated healthy volunteers. *mBio* **15**, e0237223 (2024). 38193710 10.1128/mbio.02372-23 PMC10865821

[90] Johansson, B. E., Bucher, D. J. & Kilbourne, E. D. Purified influenza virus hemagglutinin and neuraminidase are equivalent in stimulation of antibody response but induce contrasting types of immunity to infection. *J. Virol.* **63**, 1239–1246 (1989). 2915381 10.1128/jvi.63.3.1239-1246.1989 PMC247820

[91] Krammer, F. The human antibody response to influenza A virus infection and vaccination. *Nat. Rev. Immunol.* **19**, 383–397 (2019). 30837674 10.1038/s41577-019-0143-6

[92] Janssens, Y. et al. The role of cell-mediated immunity against influenza and its implications for vaccine evaluation. *Front. Immunol.* **13**, 959379 (2022). 36052083 10.3389/fimmu.2022.959379 PMC9424642

[93] van Altena, S. E. et al. Bovine natural antibodies in antibody-dependent bactericidal activity against Escherichia coli and salmonella typhimurium and risk of mastitis. *Vet. Immunol. Immunopathol.* **171**, 21–27 (2016). 26964714 10.1016/j.vetimm.2016.01.009

[94] Germon, P. & Martins, R. P. Immune defences of the mammary gland in dairy ruminants. *Reprod. Domest. Anim.* **58**, 4–14 (2023). 37133304 10.1111/rda.14372

[95] Boerhout, E. et al. Immunization routes in cattle impact the levels and neutralizing capacity of antibodies induced against S. aureus immune evasion proteins. *Vet. Res.* **46**, 115 (2015). 26411347 10.1186/s13567-015-0243-7 PMC4584483

[96] Ster, C. et al. Immune and experimental infection responses of dairy cows vaccinated with the combination of six Staphylococcus aureus proteins that are expressed during bovine intramammary infection and a triple adjuvant. *Vet. Immunol. Immunopathol.* **238**, 110290 (2021). 34217108 10.1016/j.vetimm.2021.110290

[97] Herry, V. et al. Local immunization impacts the response of dairy cows to *Escherichia coli* mastitis. *Sci. Rep.* **7**, 3441 (2017). 28611405 10.1038/s41598-017-03724-7 PMC5469773

[98] Boerhout, E. M. et al. The antibody response in the bovine mammary gland is influenced by the adjuvant and the site of subcutaneous vaccination. *Vet. Res.* **49**, 25 (2018). 29490692 10.1186/s13567-018-0521-2 PMC5831572

[99] Sridhar, S. et al. Cellular immune correlates of protection against symptomatic pandemic influenza. *Nat. Med.* **19**, 1305–1312 (2013). 24056771 10.1038/nm.3350

[100] Mettelman, R. C. et al. Baseline innate and T cell populations are correlates of protection against symptomatic influenza virus infection independent of serology. *Nat. Immunol.* **24**, 1511–1526 (2023). 37592015 10.1038/s41590-023-01590-2 PMC10566627

[101] Rainard, P., Foucras, G. & Martins, R. P. Adaptive cell-mediated immunity in the mammary gland of dairy ruminants. *Front. Vet. Sci.* **9**, 854890 (2022). 35464360 10.3389/fvets.2022.854890 PMC9019600

[102] Dong, C. et al. Enhancing cross-protection against influenza by heterologous sequential immunization with mRNA LNP and protein nanoparticle vaccines. *Nat. Commun.* **15**, 5800 (2024). 38987276 10.1038/s41467-024-50087-5 PMC11237032

[103] Cebron, N. et al. Th17-related mammary immunity, but not a high systemic Th1 immune response is associated with protection against E. coli mastitis. *NPJ Vaccines* **5**, 108 (2020). 33298970 10.1038/s41541-020-00258-4 PMC7686320

[104] Benedictus, L. et al. Immunization of young heifers with staphylococcal immune evasion proteins before natural exposure to *Staphylococcus aureus* induces a humoral immune response in serum and milk. *BMC Vet. Res.* **15**, 15 (2019). 30616609 10.1186/s12917-018-1765-9 PMC6323680

[105] Yarnall, M. et al. Identifying and addressing barriers and opportunities for bovine respiratory disease complex vaccination: a consensus paper on practical recommendations for best practise vaccination. *Front. Vet. Sci.* **11**, 1368060 (2024). 38645648 10.3389/fvets.2024.1368060 PMC11027935

[106] Shi, J., Zeng, X., Cui, P., Yan, C. & Chen, H. Alarming situation of emerging H5 and H7 avian influenza and effective control strategies. *Emerg. Microbes Infect.* **12**, 2155072 (2023). 36458831 10.1080/22221751.2022.2155072 PMC9754034

